# Microsatellite Markers Reveal Genetic Diversity and Relationships within a Melon Collection Mainly Comprising Asian Cultivated and Wild Germplasms

**DOI:** 10.1155/2019/7495609

**Published:** 2019-02-11

**Authors:** Jianbin Hu, Luyin Gao, Yanbin Xu, Qiong Li, Huayu Zhu, Luming Yang, Jianwu Li, Shouru Sun

**Affiliations:** College of Horticulture, Henan Agricultural University, Zhengzhou 450002, China

## Abstract

Melon,* Cucumis melo* L., is an important horticultural crop with abundant morphological variability, but the genetic diversity and relationships within wild and cultivated melons remain unclear to date. In this study, thick-skinned (TC) (cultivated subspecies* melo*), thin-skinned (TN) (cultivated subspecies* agrestis*), and wild accessions were analyzed for genetic diversity and relationships using 36 microsatellite markers. A total of 314 alleles were detected with a mean allelic number of 8.72 and polymorphism information content of 0.67. Cluster analysis of the accessions resulted in four distinct clusters (I, II, III, and IV) broadly matching with the TC, TN, and wild groups. Cluster I contained only two Indian wild accessions. Cluster II was consisted of 49 South Asian accessions, 34 wild accessions, and 15 TN accessions. Cluster III was a typical TC group including 51 multiorigin TC accessions and one wild accession. The remaining 88 accessions, including 75 TN accessions, 6 wild accessions, and 7 TC accessions, formed the cluster IV, and all the TN and wild accessions in this cluster were from China. These findings were also confirmed by Principal component analysis and STRUCTURE analysis. The South Asian subspecies* agrestis* accessions, wild and cultivated, had close genetic relationships with a distinctive genetic background. Chinese wild melons showed closeness to cultivated subspecies* agrestis* landraces and could be a return from the indigenous cultivated melons. The AMOVA and pairwise F statistics (*F*_ST_) presented genetic differentiation among the three groups, with the strongest differentiation (*F*_ST_ = 0.380) between TC and TN melons. These results offer overall information on genetic diversity and affiliations within a variety of melon germplasms and favor efficient organization and utilization of these resources for the current breeding purpose.

## 1. Introduction

Melon (*Cucumis melo* L., 2n=2x=24) is an economically important horticultural crop widely distributed in tropical and subtropical areas. This species is highly diverse in morphology, particularly for the fruits, leading to its multiple applications. In China, the sweet fruits of melon are conventionally consumed as a dessert (called “Tiangua”) and some medium-size nonsweet fruits as vegetables (called “Caigua”), whereas some large-size nonsweet fruits are commonly used as animal fodder. Also, the abundant diversity in this species attracts a number of studies concerning phylogenetics and taxonomy [1–5]. Although there appear to be some taxonomic methods and several of them have been in controversy or contradiction, an intraspecific taxonomy in* C. melo* proposed firstly by Pitrat [3] is now generally accepted. This taxonomic criterion divides* C. melo* into two subspecies on the basis of ovary pubescence,* melo* and* agrestis*. Mostly, subspecies* melo *bears comose ovaries and subspecies* agrestis* has ovaries with glabrous skins or short hairs. Subspecies* melo*, conventionally known as thick-skinned (TC) melon in China, is characterized by large or medium fruits and grown widely around the world, while subspecies* agrestis*, also called thin-skinned (TN) melon, carries smaller fruits and is limited in East Asia, especially in China [6]. The TC and TN melons simply refer to the cultivated forms excluding the wild or feral accessions.

Wild melons or the feral forms are mainly found in the centers of origin, Africa and South Asia [7, 8]. These wild accessions are commonly considered as subspecies* agrestis* but not assigned to the specific varietas. Most wild forms have small leaves and flowers and carry small and oval fruits with thin flesh and small-size seeds [8]. Morphological differences are easily distinguished between cultivated and wild melons; however, the genetic differences at DNA level between them still remain unclear. In addition, most of the available wild melons are found in the Indian subcontinent, and whether they act as the pioneers or ancestors of the modern cultivated melons lacks sufficient evidences yet. Clarification of these issues depends not only on the phenotypic statistics but also on the genotypic data of different accession types.

Genetic diversity and relationships of* C. melo *accessions have received an enormous amount of studies [9–14], most of which focused on a certain melon group (mostly subspecies* melo*) or the accessions from a certain region. Using different marker systems, several studies analyzed the genetic diversity of melon accessions including wild melons but with a limited accession size; most of the wild accessions were found to be more close to subspecies* agrestis* [15–17]. In this study, we aimed to analyze the genetic diversity and relationships of cultivated and wild melon accessions mainly from an Asian collection using a set of 36 core microsatellite markers. These melon accessions were collected from the probable origin regions of* C. melo* ensuring an exact examination from the analysis. The information from our results will favor dissecting the lineage relationship of various melon groups and promote the utilization of these diverse plant resources.

## 2. Materials and Methods

### 2.1. Plant Materials

A total of 191 melon accessions were used in this study (Supplemental Table 1), of which 90 were TN accessions, 58 were TC accessions, and 43 were wild accessions. All the accessions were classified into 133 subspecies* agrestis* accessions (TN and wild types) and 58 subspecies* melo* accessions and covered eight countries in the world including the major origin regions of melon, such as India, Iran, Turkey, and China. A majority of the accessions (137, 71.73%) were landraces and the remaining were commercial cultivars (11, 5.76%) and wild accessions (43, 22.51%). Among them, 88 were requested from the National Mid-term Genebank for watermelon and melon (Zhengzhou, China), 86 were from USDA-ARS National Plant Germplasm System, and the remaining 17 were from the Research Group of Watermelon and Melon at Henan Agricultural University.

### 2.2. Microsatellite Marker Genotyping

Genomic DNA was extracted from young leaf samples of all the accessions using a CTAB procedure described by Doyle and Doyle [18]. SSR markers were used for genotyping. Initially, we collected 300 SSR primer pairs from the published reports [19, 20] and then developed 70 SSR markers from the melon lines DHL92 and TopMark genome assembly to fill up the marker gaps in the melon chromosomes. These markers were screened using 10 diverse accessions (four subspecies* agrestis* accessions, three subspecies* melo* accessions, and three wild accessions). Also, this screening took into account even distribution across the melon chromosomes. Finally, a set of 36 high-polymorphism SSR markers were obtained, with each chromosome containing three markers at the top, medium, and bottom of the chromosomes. As a result, 15 SSRs were coming from the report of Zhu* et al*. [19], 13 were from the consensus linkage map of Diaz* et al*. [20], and the remaining 8 were newly developed by our research group according to the method of Zhu* et al*. [19]. Detailed information of the 36 markers is listed in Supplementary Table 2.

PCR amplification was carried out in a Thermal Cycler (BIORAD C1000™) with the reaction system and amplification program being same to the report of Wang* et al*. [14]. The amplification products were analyzed by electrophoresis on 6% polyacrylamide gels (19: 1 acrylamide: bis). The band patterns were visualized by silver staining and recorded with a digital camera. The band sizes for each locus were estimated by reference to a DNA ladder (pUC19 DNA/*Msp*l marker, Sangon Biotech, Shanghai).

### 2.3. Data Analysis

All the markers were scored as codominant data according to the amplicon size. This resulted in a genotypic matrix that was used to calculate the genetic parameters with the software PowerMarker v3.51 [21], i.e., the number of observed alleles (Na) and effective alleles (Ne), Shannon's information index (I), observed (Ho), and expected (He) heterozygosity. Polymorphic information content (PIC) for each marker was calculated using an online program PICcalc [22] that adopted the formula described by Botstein* et al*. [23].

To analyze the genetic diversity and relationship of the melon accessions, the genotypic data were imported into the software MEGA6 [24] to construct a neighbor-joining (NJ) dendrogram. Also, confirmation of the genetic relationships among the accessions was performed using a principal component analysis (PCA) implemented in the software NTSYSpc 2.20e [25] and a model-based program available in STRUCTURE 2.3.1 [26]. The former resulted in a two-dimensional PCA plot showing clustering patterns of the accessions by performing the DCENTER and EIGEN modules in NTSYSpc 2.20e, and the latter offered the clusters for all the K values. The optimal K values (the number of subpopulations in the whole collection) was determined using Markov Chain Monte Carlo (MCMC) algorithm in STRUCTURE HARVESTER. Briefly, each of the probable K was run 10 times with K=1 to 10, and the length of burn-in period was separately set at 10,000 and 100,000 MCMC repeats after burn-in with an admixture and allele frequency correlated model. The optimal K was determined by the log probability of data [Lnp(D)] from the output and the Evanno's ΔK between successive K values [27].

Genetic differentiation among the different groups was measured by calculating pairwise F statistics (*F*_ST_), genetic distance [28], and analysis of molecular variance (AMOVA) using GeneAlEx 6.5 [29].

## 3. Results

### 3.1. Characterization of Microsatellite Marker Polymorphism

All the 36 SSR markers produced clear band patterns, revealing single-locus variation among the melon accessions. Five genetic parameters (Na, Ne, Ho, He, and PIC) were calculated for the 36 markers estimated from the 191 melon accessions, as shown in Table 1. In total, 314 alleles were detected varying from 5 (SSR020162, SSR020947, SSR029474, and SSR038372) to 18 (SSR013487) with a mean of 8.72. Ne, an important parameter to measure genetic diversity in a finite population, averaged 3.88 ranging from 1.94 (HNM41) to 7.67 (CMAGN75). No heterozygosity deficiency was observed in the accession collection; the Ho values were quite low (<0.20) at the loci with a mean of 0.08. He means expected heterozygosity in a certain population and averaged 0.72 in the accession collection. The highest (0.87) and lowest (0.56) He values were observed for CMAGN75 and SSR038372, respectively. For each locus, He value was much higher than Ho value, revealing a high homozygosity at the given loci among the accessions. PIC is generally used for characterization of marker polymorphism and the values ranged between 0.45 (HNM41) and 0.86 (CMAGN75) (mean=0.68) in the accession collection. All the genetic parameters revealed a high level of polymorphism for the 36 markers, favoring the establishment of the genetic affiliations within the melon collection.

### 3.2. Establishment of Genetic Relationship for the Accession Collection

**Figure 1:**
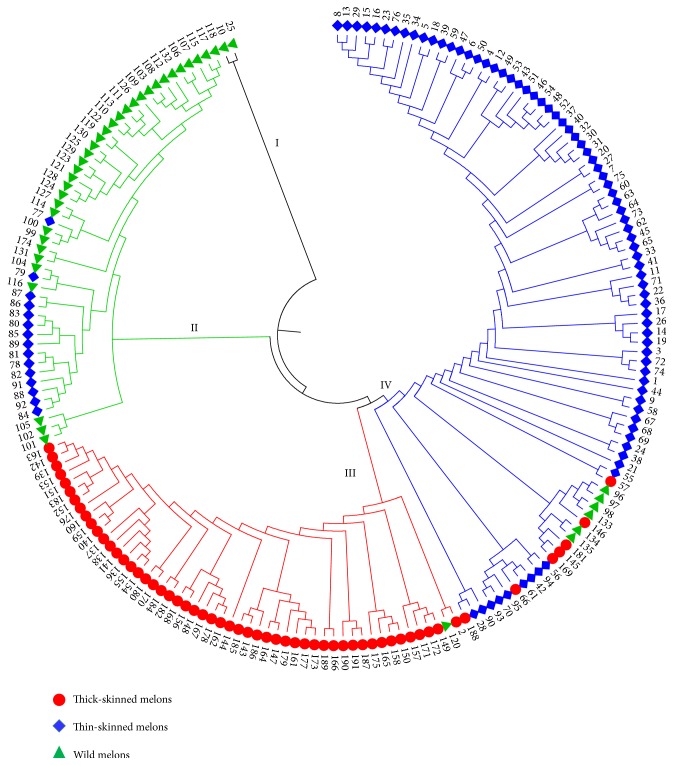
A neighbor-joining dendrogram showing the genetic affiliations of the 191 melon accessions. All the accessions were divided into four clusters (I, II, III, and IV). Numbers indicate the accession codes as listed in Supplementary Table 1.

With the SSR genotypic data, a NJ dendrogram (Figure 1) was constructed based on Nei's similarity coefficients [30] showing the genetic relationship among the accessions. The dendrogram clustered the 191 accessions into four distinct clusters (I, II, III, and IV). Cluster I contained only two Indian wild accessions (No. 10 and 25), which were highly diverse and distinguished from the other accessions. Cluster II was consisted of 34 wild accessions and 15 TN landraces (*momordica* accessions) with the pairwise genetic distances (GDs) varying from 0.23 to 0.78 (mean = 0.63). Most of the accessions (32 wild accessions and 14 TN landraces) in this cluster derived from India and only three from Maldives, two wild accessions (No. 99 and 100) and one TN landrace (No. 77). Clearly, the wild and TN accessions in this cluster from the two adjacent countries had close lineages. Cluster III was a typical TC group (GDs ranging from 0.20 to 0.67, mean = 0.54), containing 51 TC accessions (subspecies* melo*) and one wild accession (No. 120). These accessions in this cluster were morphologically diverse belonging to at least five varietas suggested by Pitrat [3], such as* cantalupensis*,* reticulatus*,* inodorus*,* ameri*, and* chandalak*, and also, their origins covered a wide geographical distribution (China, India, Tunisia, Japan, Afghanistan, and Iran). Although these subspecies* melo* landraces in cluster III derived from different regions, they had close genetic relationships and similar genetic backgrounds, probably implying their same origin. The wild accession in this cluster III (No. 120) was an exception since it was clustered together with the subspecies* melo* accessions, and therefore it could involve in gene exchange with the subspecies* melo* plants.

The remaining 88 accessions formed cluster IV that covered both the two subspecies (TC, TN, and wild groups), mainly representing by East Asian TN melons (i.e., 75 subspecies* agrestis *accessions). The TN accessions in this cluster included 48* chinensis* accessions, 13* conomon* accessions, 7* makuwa* accessions, 6* momordica* accessions and one* acidulous* accession, most of which derived from China. Except for the TN accessions, cluster IV possessed 7 TC accessions (No. 57, 66, 145, 146, 169, 181, and 188) and 6 wild accessions (No. 96, 97, 98, 133, 134, and 135). This fact indicated close affiliations of these accessions. The seven TC accessions were clustered into this cluster, perhaps due to the introgression of the subspecies* agrestis* lineages during their domestication processes. It should be noted that six wild accessions were also included in this cluster, in contrast to most of the wild accessions assigned to cluster II. Of these wild accessions, 4 were from China and the other two from Costa Rica and the US, which were markedly different from the wild accessions in clusters I and II with South Asian origin. A lower level of variation was observed for cluster IV with the GDs ranging from 0.18 to 0.72 (mean = 0.45), indicating a comparatively narrow genetic basis in this cluster.

**Figure 2:**
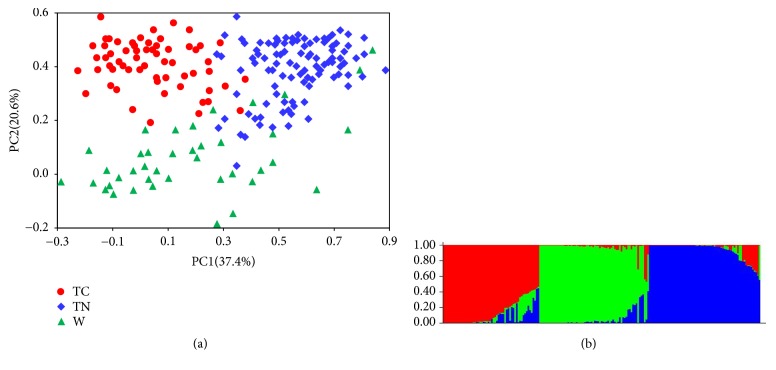
Genetic structure of the 191 melon accessions revealed by principal component analysis (PCA) (a) and STRUCTURE analysis (b). The symbols and colors for the accessions correspond to those of the Figure 1. TC, TN, and W represent thick-skinned, thin-skinned, and wild accessions, respectively.

Furtherly, two methods, principal component analysis (PCA) and STRUCUTRE analysis, were used to offer an alternative view of the relationships within the accession collection. On the PCA dendrogram (Figure 2(a)), all the melon accessions, which were labelled with different symbols and colors according to the accession classification, tended to form three clusters, i.e., the red, blue, and green regions. Most of the TC accessions were positioned to the red region while the TN accessions were mainly to the blue region. These two regions were not separated absolutely because some TN and TC accession (e.g., No. 28, 42, 61, 66, 57, 146, 181, and 188) were mixed together. The wild accessions (green triangles) were scattered across a wide area, even some (e.g., No. 96, 97, 98, 133, 134, and 135) seeping into the blue region. This implied the complex genetic background of the wild forms. Similarly, the STRUCUTRE analysis positioned all the accessions into three subpopulations (Figure 2(b)), which represented TC (red), TN (blue) and wild (green) groups, respectively. Obviously, the three methods gave similar results on positioning of the melon accessions.

### 3.3. Comparison of Genetic Diversity for Wild, TC, and TN Groups

**Figure 3:**
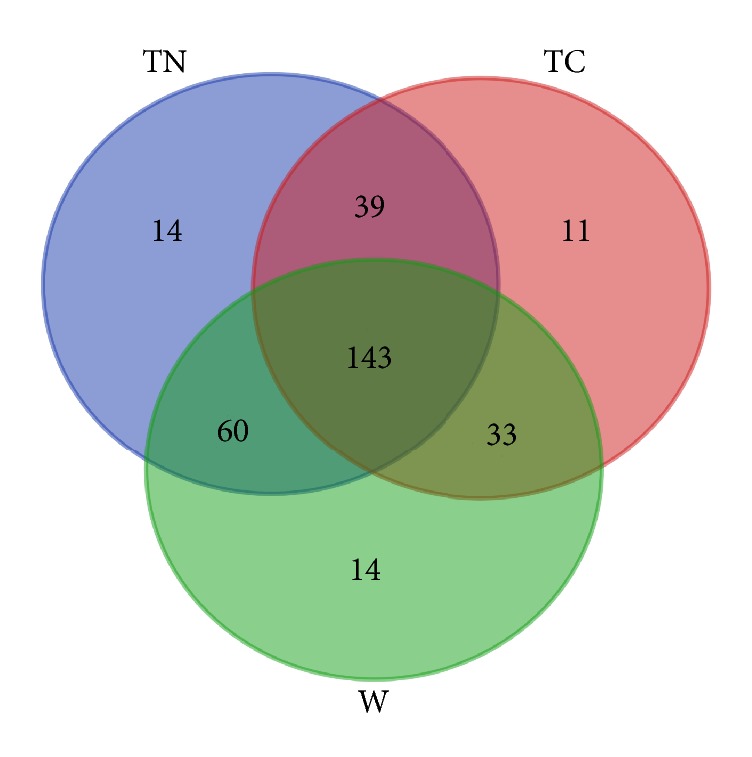
A Venn diagram showing the number of alleles specific to a certain group or shared by different groups. TC, TN, and W mean the thick-skinned, thin-skinned, and wild groups, respectively.

Since distinct divergences were found among the different groups, the six genetic parameters (Na, Ne, Ho, He, I, and PIC) were computed for each group to compare their diversity levels. As shown in Table 2, TN and wild groups had more Na than TC group demonstrating a higher level of allelic polymorphism. Wild group showed the highest values of Ne (4.08) and He (0.72) indicating a wide heterogenicity at the genome level. The sample size of wild accessions was the smallest among the three groups; however, the three parameters (He, I, and PIC) that reflect diversity level revealed the highest values in wild group and verified its abundant diversity. According to the parameter values (Ne, He, and PIC) in Table 2, TC group was more slightly diverse than TN group. In addition, the alleles that are specific to a certain group and the ones shared by the two or more groups were shown in Figure 3. Both TN and wild groups had 14 group-specific alleles, whereas TC group possessed such 11 genes. Certainly, the shared alleles among the groups accounted for the main part, 143 alleles shared by all the three groups and 33–60 alleles by each two groups. Combination of the findings from the genetic parameters and group-specific alleles demonstrated the highest level of diversity present within wild accessions, following by cultivated subspecies* melo* and* agrestis* accessions.

### 3.4. Examination of Genetic Differentiation for the Collection

**Figure 4:**
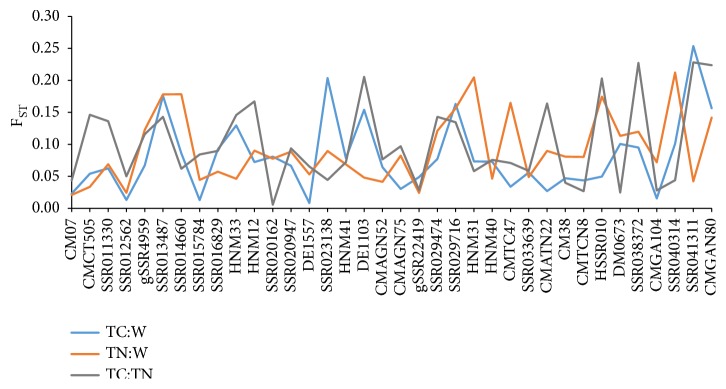
Pairwise *F*_ST_ values among the thick-skinned (TC), thin-skinned (TN), and wild (W) accessions at the 36 marker loci.

To analyze the genetic differentiation of the collection, AMOVA was conducted using accession groups and geographic origins as sources of variation, and showed that 28.70% of the total variation was attributed to the differentiation between groups and 30.44% was to the differentiation between geographic regions (Table 3). The highest percentage (37.42%) occurred among the accessions while the variation within accessions was quite low with the percentage of 3.44%. Given that the geographic origins were basically related to the melon classification (Supplemental Table 1), the accession type was an important factor leading to genetic differentiation. Also, the differentiation among the three groups was measured by pairwise *F*_ST_ and Nei's genetic distance (Table 4). Each of the pairwise *F*_ST_ among the three groups was higher than 0.25, a threshold for existence of a very high level of genetic differentiation suggested by Wright [31]. That indicated that each of the melon groups was clearly differentiated from the others. The highest pairwise *F*_ST_ value of 0.380 was found between the TC and TN groups and displayed a strong genetic differentiation. This was also confirmed by the maximum genetic distance (0.102) between the TC and TN groups. The minimum genetic distance (0.083) occurred between TC and wild groups. Of the 36 marker loci, 15 showed clear differentiation among the groups with the pairwise *F*_ST_ value of >0.15 (Figure 4), such as SSR013487, SSR014660, HNM33, HNM12, SSR023138, DE1103, SSR029716, HNM31, CMTC47, CMATN22, HSSR010, SSR038372, SSR040314, SSR041311, and CMGAN80. These loci could reflect evolutionary forces (e.g., artificial selection) affecting domestication of cultivated melons.

## 4. Discussion

A diversity of plant germplasms is valuable resources for present and future commercial producers and researchers; they can be used for breeding of new cultivars to meet the demand for food and studying the origin, evolution, and taxonomy of plant species. Melon is such a horticultural crop with abundant diversity. During the past decades, it earned worldwide attentions in scientific research (e.g., developmental biology and genetics) [32, 33] and agricultural production as its yield of fresh fruits frequently entered the top ten of the main fruits in the world (FAO Statistics from 2007-2016, http://www.fao.org/faostat/en/#data/QC).

*C. melo* has a large number of morphotypes, cultivated and wild [8]; cultivated melons scatter around the world and wild melons are mainly concentrated in North Africa and South Asia [34, 35]. In China, cultivated melons are commonly distinguished into TC and TN groups, which respectively correspond to the two subspecies,* melo* and* agrestis*. TN melons (subspecies* agrestis* group) are special morphotypes mainly distributed in East Asia, especially in central and eastern China, the important diversity center of subspecies* agrestis* in the world [14, 35]. This kind of melons has attracted wide attentions these years for the striking characteristics, such as adversity tolerance, early maturity, vigorous growth and good fruit set. TC melons (subspecies* melo* group), having a large number of commercial cultivars in the world, are the main cultivated forms. To utilize efficiently these melon resources in modern agriculture, we adopted 36 core microsatellite markers to examine the genetic diversity and relationships present within an Asian melon collection. Our assays detected 314 alleles (mean=8.72) with a mean PIC value of 0.67, showing a high level of variation in the melon genome. The mean allelic number and PIC value were much higher than those of available reports with the experimental materials being a certain group or from a certain region [11–14]. This could be due to the diverse accessions used in the present study. Gao* et al*. [36] expanded the sample size to 471 melon accessions and detected a higher level of variation, a mean allelic number of 9.0 per SSR locus and PIC value of 0.68.

To date, genetic diversity and relationships in* C. melo* have been frequently reported mainly focusing on cultivated accessions. These results showed that subspecies* melo* accessions were obviously distinguished from subspecies* agrestis* accessions [4, 36–39], implying an existence of genetic divergence at the subspecies level. Also, several other reports, which involved some wild melon accessions mainly from Africa, India, and America, showed that African and Indian wild accessions were close to* conomon*,* chito*,* dudaim*, and* momordica*, but far from American and European* cantalupensis* and* inodorus *[2, 15–17]. Based on the combination of phenotypic characters and molecular marker data, American wild melons also showed genetic affinities to Asian subspecies* agrestis *[17]. These American wild accessions were assumed to be the introduction from India. As expected, the wild melon accessions in the present research, especially for Indian wild accessions, were highly diverse (Table 2) and most of them were distinctly different from the cultivated accessions (TC and Chinese TN accessions) (Figures 1 and 2; Table 4). Indian subspecies* agrestis* landraces were clustered closely with the wild accessions showing their close genetic relationship, as a probable result of the frequent gene exchanges (e.g., mutual pollination). From the records of USDA-ARS National Plant Germplasm System (Data not shown), these Indian subspecies* agrestis* accessions have similar morphological features to the wild species and are probably old indigenous landraces or semiwild forms. Since there are a variety of wild melons and semiwild forms or landraces in India, it is reasonable to assume that Indian subcontinent acts as the center of origin of this crop [7]. This view is supported by the present results as well as the previous reports. Interestingly, the Chinese wild accessions were genetically close to the cultivated subspecies* agrestis* accessions (Chinese TN accessions) in group IV. The same finding was also described in our previous study [40]. A view proposed by Pitrat [8] is that the “wild melon” from the New World is not true wild form but a return to a wild status from cultivated melons. Chinese wild melons are likely to be such a case. There are numerous wild melons scattering across Central China, particularly in Henan province, which are customarily called “Mapao”, a common weedy fruit in crop field. It bears a large number of small-size fruits (~ 20 g) but with domesticated characters, e.g., yellow skin, sweet flesh, and aroma. This kind of “wild melons” could be an escape from indigenous cultivated melons, probably the subspecies* agrestis*. Therefore, East Asian subspecies* agrestis* melons probably originated in Indian subcontinent and were intensively domesticated in Central China, as the indigenous wild melons could not be their pioneers or ancestors. The similar view has been suggested in several studies that East Asian subspecies* agrestis* melons benefited from Indian introduction (perhaps via Myanmar, Laos, and eastern China; ~100 BC) and were domesticated in Huang-Huai-Hai plain in China [37, 41].

The strongest differentiation occurred between TC and TN melons (*F*_ST_ = 0.380); such a high level of differentiation could be supported by a fact that TC and TN accessions belong to two different subspecies,* melo* and* agrestis* [3, 5]. Also, the subspecies divergence in* C. melo* have been reported in several studies [2, 36–38], although the accessions of the two subspecies were of multiple-origin. As worldwide-distribution cultivated forms, subspecies* melo* (TC melons) were initially considered to originate from Africa [1, 34, 35] but the later researches evidence their Asian origin [7]. According to the taxonomy of Pitrat [3], subspecies* melo* possesses 11 varietas and is richer in morphological diversity than subspecies* agrestis* (containing 5 varietas), as also evidenced from our result (Table 2). This could be due to undergo different selection patterns; subspecies* melo* melons underwent selections over the worldwide regions while selection of subspecies* agrestis* melons was mainly restricted in Central China.

In the present research, we used SSR markers separated by polyacrylamide gel. It would be interesting to compare phylogenetic relationships among studied melon accessions using another method like capillary electrophoresis or even other molecular approaches including SNP or DArT markers. Such new researches can confirm or show a conflict with current study for the genetic clustering and it will be the next step for the investigation.

## 5. Conclusions

Genetic diversity and relationship are crucial for plant breeding as they determine the efficient utilization of the genetic materials and selection of potential parents. Accurate measurement of genetic diversity and relationship present within an accession collection relies on the molecular markers (e.g., SSR) with stability and even distribution across the genome. The present study revealed an existence of distinct population structure in 191 melon accessions. Indian wild accessions, revealing a close relationship to the local subspecies* agrestis* landraces, had a high level of variation and were distinguished from the cultivated accessions, subspecies* melo* and subspecies* agrestis*. Chinese wild melons also showed close lineages to the local subspecies* agrestis* accessions. Genetic differentiations occurred among wild accessions, cultivated subspecies* melo,* and subspecies* agrestis* accessions; the strongest differentiation was between cultivated subspecies* melo* and* agrestis* accessions indicating a subspecies-level divergence. The information of the genetic diversity and relationships among the cultivated and wild melons will be helpful for efficient organization and utilization of these genetic materials in current breeding programs.

## Figures and Tables

**Table 1:** Statistics of genetic variation as measured for 36 SSRs estimated from 191 melon accessions.

Marker	Na	Ne	Ho	He	PIC

CM07	7	5.83	0.04	0.83	0.60
CMCT505	8	3.89	0.09	0.75	0.71
SSR011330	9	3.63	0.11	0.73	0.69
SSR012562	9	3.39	0.12	0.71	0.68
gSSR4959	6	1.95	0.03	0.49	0.46
SSR013487	16	3.41	0.16	0.71	0.69
SSR014660	14	4.75	0.05	0.79	0.78
SSR015784	6	3.69	0.11	0.73	0.68
SSR016829	9	3.97	0.14	0.75	0.71
HNM33	10	4.32	0.10	0.77	0.74
HNM12	11	5.92	0.11	0.83	0.81
SSR020162	5	2.46	0.05	0.60	0.54
SSR020947	5	2.31	0.06	0.57	0.50
DE1557	12	5.25	0.03	0.81	0.79
SSR023138	8	4.20	0.16	0.76	0.74
HNM41	7	1.94	0.13	0.48	0.45
DE1103	7	2.40	0.09	0.58	0.55
CMAGN52	9	3.54	0.02	0.72	0.70
CMAGN75	13	7.67	0.05	0.87	0.86
gSSR22419	6	3.15	0.04	0.68	0.63
SSR029474	5	4.21	0.03	0.76	0.72
SSR029716	9	4.47	0.02	0.78	0.75
HNM31	8	4.04	0.09	0.75	0.71
HNM40	8	4.11	0.15	0.76	0.73
CMTC47	8	3.08	0.18	0.68	0.64
SSR033639	8	4.70	0.02	0.79	0.76
CMATN22	7	3.32	0.07	0.70	0.65
CM38	9	4.81	0.11	0.79	0.76
CMTCN8	10	2.56	0.05	0.61	0.58
HSSR010	9	5.16	0.05	0.81	0.79
DM0673	12	6.08	0.11	0.84	0.82
SSR038372	5	2.29	0.05	0.56	0.47
CMGA104	9	3.68	0.05	0.73	0.69
SSR040314	10	3.51	0.06	0.72	0.67
SSR041311	11	3.56	0.04	0.72	0.70
CMGAN80	9	2.54	0.11	0.61	0.58
Mean	8.72	3.88	0.08	0.72	0.68

Na: the number of observed alleles.

Ne: the number of effective alleles.

Ho: observed heterozygosity.

He: expected heterozygosity.

PIC: polymorphic information content.

**Table 2:** Comparison of genetic diversity for the thick-skinned (TC), thin-skinned (TN), and wild (W) groups.

Accession group	Na	Ne	Ho	He	I	PIC

TC	226	2.93	0.04	0.63	1.26	0.58
TN	256	2.87	0.07	0.59	1.26	0.55
W	250	4.08	0.18	0.72	1.51	0.67

Na: the number of observed alleles.

Ne: the number of effective alleles.

Ho: observed heterozygosity.

He: expected heterozygosity.

I: Shannon's information index.

PIC: Polymorphic information content.

**Table 3:** Molecular analyses of variance (AMOVA) among the accession groups and origin regions.

Source of variation	df	Variance components	Percentage of variation	*P* value

Among groups	2	58.03	28.70	<0.01
Among regions	2	61.54	30.44	<0.01
Among individuals	176	75.67	37.42	<0.01
Within individuals	191	6.96	3.44	<0.01

Groups were defined by the thick-skinned, thin-skinned, and wild accessions.

Regions were defined by South Asia (India and Maldives), East Asia (China, Malaysia, and Japan), West Asia (Iran and Turkey), and Africa (Tunisia).

**Table 4:** Pairwise estimates of genetic differentiation among the three accession groups using pairwise *F*_ST_ (above the diagonal) and Nei's genetic distance (below the diagonal). Permutation tests confirmed that all the *F*_ST_ values were significant at *P* < 0.01.

Accession group	TC	TN	W

TC	—	0.380	0.293
TN	0.102	—	0.319
W	0.083	0.100	—

TC, TN, and W mean the thick-skinned, thin-skinned, and wild groups.

## Data Availability

The data used to support the findings of this study are included within the article.

## Supplementary Materials

Supplementary Materials Table 1: descriptions of the 191 melon accessions used in the present study. Table 2: the information of the 37 SSR markers used in the present study.
