# Supplementary material for: Microsatellite Markers Reveal Genetic Diversity and Relationships within a Melon Collection Mainly Comprising Asian Cultivated and Wild Germplasms
Source: Biomed Res Int. 2019 Feb 11;2019:7495609. doi: 10.1155/2019/7495609 (PMC6388322; doi:10.1155/2019/7495609)
Supplement: Supplementary Materials — Table 1: descriptions of the 191 melon accessions used in the present study. Table 2: the information of the 37 SSR markers used in the present study. [file 7495609.f1.docx]

**Table S1** Descriptions of the 191 melon accessions used in the present study.

| No. | Accession | Subspecies | varietas | Type | Growth Status | Origin | Seed source | Cluster |
| --- | --- | --- | --- | --- | --- | --- | --- | --- |
| 1 | Greenskin-3 | *agrestis* | *chinensis* | TN | L | Sinkiang, China | NMGWM | IV |
| 2 | Geperrial | *melo* | *cantalupensis* | TC | L | Sinkiang, China | NMGWM | III |
| 3 | Lougua | *agrestis* | *chinensis* | TN | L | Henan, China | NMGWM | IV |
| 4 | Baishami | *agrestis* | *chinensis* | TN | L | Jilin, China | RGWM | IV |
| 5 | Bingtangguan | *agrestis* | *chinensis* | TN | L | Henan, China | NMGWM | IV |
| 6 | Huapimelon | *agrestis* | *chinensis* | TN | L | Jiangsu, China | NMGWM | IV |
| 7 | Kusong | *agrestis* | *makuwa* | TN | L | Malaysia | NMGWM | IV |
| 8 | Yinhui | *agrestis* | *makuwa* | TN | L | Taiwan, China | NMGWM | IV |
| 9 | Xiaocuigua | *agrestis* | *chinensis* | TN | L | Hebei, China | NMGWM | IV |
| 10 | PI614410 | *agrestis* | ND | ND | W | India | NGRP | I |
| 11 | TM-1 | *agrestis* | *chinensis* | TN | L | Guangxi, China | RGWM | IV |
| 12 | Yiduhuangyin-1 | *agrestis* | *chinensis* | TN | L | Shandong, China | NMGWM | IV |
| 13 | Yinxuanhuangzi | *agrestis* | *chinensis* | TN | L | Henan, China | NMGWM | IV |
| 14 | Kangua | *agrestis* | *chinensis* | TN | L | Jiangsu, China | NMGWM | IV |
| 15 | Jiangxiligua | *agrestis* | *chinensis* | TN | L | Jiangxi, China | NMGWM | IV |
| 16 | Pingguoqing | *agrestis* | *conomon* | TN | L | Jiangxi, China | NMGWM | IV |
| 17 | Tiancaigua | *agrestis* | *conomon* | TN | L | Zhejiang, China | NMGWM | IV |
| 18 | Yiwohou | *agrestis* | *chinensis* | TN | L | Shanxi, China | NMGWM | IV |
| 19 | Shitiaoxian | *agrestis* | *chinensis* | TN | L | Shaanxi, China | NMGWM | IV |
| 20 | Xiguahun | *agrestis* | *makuwa* | TN | L | Henan, China | NMGWM | IV |
| 21 | PI614323 | *agrestis* | *momordica* | TN | L | India | NGRP | IV |
| 22 | Qingpicui | *agrestis* | *chinensis* | TN | L | Liaoning, China | NMGWM | IV |
| 23 | Jindaozi | *agrestis* | *chinensis* | TN | L | Liaoning, China | NMGWM | IV |
| 24 | Sweet Banana | *agrestis* | *conomon* | TN | L | Liaoning, China | NMGWM | IV |
| 25 | PI614412 | *agrestis* | ND | ND | W | India | NGRP | I |
| 26 | Hebei melon | *agrestis* | *chinensis* | TN | L | Hebei, China | NMGWM | IV |
| 27 | Jinbachi | *agrestis* | *chinensis* | TN | L | Inner Mongolia, China | NMGWM | IV |
| 28 | Putian-1 | *agrestis* | *makuwa* | TN | L | Fujian, China | NMGWM | IV |
| 29 | Hongzicui | *agrestis* | *chinensis* | TN | L | Henan, China | NMGWM | IV |
| 30 | Huadaozi | *agrestis* | *chinensis* | TN | L | Liaoning, China | NMGWM | IV |
| 31 | Ice melon | *agrestis* | *chinensis* | TN | L | Henan, China | NMGWM | IV |
| 32 | Huhe-3 | *agrestis* | *conomon* | TN | L | Inner Mongolia, China | NMGWM | IV |
| 33 | Miangua | *agrestis* | *chinensis* | TN | L | Anhui, China | NMGWM | IV |
| 34 | Xiaolenggua | *agrestis* | *chinensis* | TN | L | Jiangsu, China | NMGWM | IV |
| 35 | Yangshuigua | *agrestis* | *chinensis* | TN | L | Jiangsu, China | NMGWM | IV |
| 36 | Shushugua | *agrestis* | *chinensis* | TN | L | Shanxi, China | NMGWM | IV |
| 37 | Xiehuatian | *agrestis* | *chinensis* | TN | L | Henan, China | NMGWM | IV |
| 38 | Green Banana | *agrestis* | *chinensis* | TN | L | Heilongjiang, China | NMGWM | IV |
| 39 | Balixiang | *agrestis* | *chinensis* | TN | L | Jilin, China | NMGWM | IV |
| 40 | Jinhui | *agrestis* | *chinensis* | TN | L | Taiwan, China | NMGWM | IV |
| 41 | Optimum | *agrestis* | *makuwa* | TN | L | Heilongjiang, China | NMGWM | IV |
| 42 | Comet | *agrestis* | *chinensis* | TN | L | Sinkiang, China | NMGWM | IV |
| 43 | Shilenggou Jingua | *agrestis* | *chinensis* | TN | L | Tianjin, China | NMGWM | IV |
| 44 | Huangfei | *agrestis* | *chinensis* | TN | L | Hebei, China | NMGWM | IV |
| 45 | Miangua | *agrestis* | *chinensis* | TN | L | Shandong, China | NMGWM | IV |
| 46 | Tiebachi | *agrestis* | *conomon* | TN | L | Anhui, China | NMGWM | IV |
| 47 | Sanyehua Crisp | *agrestis* | *conomon* | TN | L | Jiangsu, China | NMGWM | IV |
| 48 | Xinyuzhimacui | *agrestis* | *conomon* | TN | L | Henan, China | NMGWM | IV |
| 49 | Elingbai | *agrestis* | *conomon* | TN | L | Hebei, China | NMGWM | IV |
| 50 | Gold-9 | *agrestis* | *makuwa* | TN | L | Japan | NMGWM | IV |
| 51 | Wanggudan | *agrestis* | *makuwa* | TN | L | Heilongjiang, China | NMGWM | IV |
| 52 | Shanghaimelon | *agrestis* | *chinensis* | TN | L | Shanghai, China | NMGWM | IV |
| 53 | White Banana | *agrestis* | *chinensis* | TN | L | Heilongjiang, China | NMGWM | IV |
| 54 | Yangjiaomi | *agrestis* | *chinensis* | TN | C | Shandong, China | NMGWM | IV |
| 55 | PI614514 | *agrestis* | *momordica* | TN | L | India | NGRP | IV |
| 56 | Bazhangqing | *agrestis* | *conomon* | TN | L | Japan | NMGWM | IV |
| 57 | PI321004-S2 | *melo* | *reticulatus* | TC | L | Taiwan, China | NMGWM | IV |
| 58 | Liujingua | *agrestis* | *chinensis* | TN | L | Shandong, China | NMGWM | IV |
| 59 | Hefeibaigua | *agrestis* | *chinensis* | TN | L | Anhui, China | NMGWM | IV |
| 60 | Green Flesh | *agrestis* | *conomon* | TN | L | Jiangsu, China | NMGWM | IV |
| 61 | Bailigua | *agrestis* | *chinensis* | TN | L | Hubei, China | NMGWM | IV |
| 62 | Liuyin | *agrestis* | *conomon* | TN | L | Henan, China | NMGWM | IV |
| 63 | Hamasu-5 | *agrestis* | *acidulus* | TN | L | Liaoning, China | NMGWM | IV |
| 64 | Xiaohuagua | *agrestis* | *chinensis* | TN | L | Henan, China | RGWM | IV |
| 65 | YangjiaosuS1 | *agrestis* | *chinensis* | TN | C | Henan, China | RGWM | IV |
| 66 | Yutiancui | *melo* | *cassaba* | TC | C | Henan, China | RGWM | IV |
| 67 | Snow sweet | *agrestis* | *chinensis* | TN | C | Henan, China | RGWM | IV |
| 68 | Cuilv | *agrestis* | *chinensis* | TN | C | Henan, China | RGWM | IV |
| 69 | Weizhi5 | *agrestis* | *chinensis* | TN | C | Henan, China | RGWM | IV |
| 70 | Baijinmi | *agrestis* | *chinensis* | TN | C | Henan, China | RGWM | IV |
| 71 | Changtiancaigua | *agrestis* | *conomon* | TN | C | Henan, China | RGWM | IV |
| 72 | Cuicaigua | *agrestis* | *conomon* | TN | C | Henan, China | RGWM | IV |
| 73 | Huangtiancui | *agrestis* | *chinensis* | TN | C | Henan, China | RGWM | IV |
| 74 | Tedaheipimiangua | *agrestis* | *chinensis* | TN | C | Henan, China | RGWM | IV |
| 75 | Xiaohuanggua | *agrestis* | *chinensis* | TN | L | Henan, China | RGWM | IV |
| 76 | Ames31282 | *agrestis* | *chinensis* | TN | L | Henan, China | NGRP | IV |
| 77 | PI536473 | *agrestis* | *momordica* | TN | L | Maldives | NGRP | II |
| 78 | PI614390 | *agrestis* | *momordica* | TN | L | India | NGRP | II |
| 79 | PI614391 | *agrestis* | *momordica* | TN | L | India | NGRP | II |
| 80 | PI614392 | *agrestis* | *momordica* | TN | L | India | NGRP | II |
| 81 | PI614393 | *agrestis* | *momordica* | TN | L | India | NGRP | II |
| 82 | PI614395 | *agrestis* | *momordica* | TN | L | India | NGRP | II |
| 83 | PI614397 | *agrestis* | *momordica* | TN | L | India | NGRP | II |
| 84 | PI614398 | *agrestis* | *momordica* | TN | L | India | NGRP | II |
| 85 | PI614399 | *agrestis* | *momordica* | TN | L | India | NGRP | II |
| 86 | PI614512 | *agrestis* | *momordica* | TN | L | India | NGRP | II |
| 87 | PI614541 | *agrestis* | *momordica* | TN | L | India | NGRP | II |
| 88 | PI614549 | *agrestis* | *momordica* | TN | L | India | NGRP | II |
| 89 | PI614465 | *agrestis* | *momordica* | TN | L | India | NGRP | II |
| 90 | PI614523 | *agrestis* | *momordica* | TN | L | India | NGRP | IV |
| 91 | PI614548 | *agrestis* | *momordica* | TN | L | India | NGRP | II |
| 92 | PI614565 | *agrestis* | *momordica* | TN | L | India | NGRP | II |
| 93 | PI164774 | *agrestis* | *momordica* | TN | L | India | NGRP | IV |
| 94 | Ames21185 | *agrestis* | *momordica* | TC | L | Turkey | NGRP | IV |
| 95 | PI614576 | *agrestis* | *momordica* | TN | L | India | NGRP | IV |
| 96 | Yetiangua | *agrestis* | ND | ND | W | Sinkiang, China | NMGWM | IV |
| 97 | Mapaogua | *agrestis* | ND | ND | W | Henan, China | RGWM | IV |
| 98 | PI406737 | *agrestis* | ND | ND | W | Costa Rica | NGRP | IV |
| 99 | PI536473 | *agrestis* | ND | ND | W | Maldives | NGRP | II |
| 100 | PI536476 | *agrestis* | ND | ND | W | Maldives | NGRP | II |
| 101 | PI614173 | *agrestis* | ND | ND | W | India | NGRP | II |
| 102 | PI614174 | *agrestis* | ND | ND | W | India | NGRP | II |
| 103 | PI614307 | *agrestis* | ND | ND | W | India | NGRP | II |
| 104 | PI614309 | *agrestis* | ND | ND | W | India | NGRP | II |
| 105 | PI614325 | *agrestis* | ND | ND | W | India | NGRP | II |
| 106 | PI614328 | *agrestis* | ND | ND | W | India | NGRP | II |
| 107 | PI614329 | *agrestis* | ND | ND | W | India | NGRP | II |
| 108 | PI614330 | *agrestis* | ND | ND | W | India | NGRP | II |
| 109 | PI614335 | *agrestis* | ND | ND | W | India | NGRP | II |
| 110 | PI614356 | *agrestis* | ND | ND | W | India | NGRP | II |
| 111 | PI614361 | *agrestis* | ND | ND | W | India | NGRP | II |
| 112 | PI614363 | *agrestis* | ND | ND | W | India | NGRP | II |
| 113 | PI614364 | *agrestis* | ND | ND | W | India | NGRP | II |
| 114 | PI614438 | *agrestis* | ND | ND | W | India | NGRP | II |
| 115 | PI614439 | *agrestis* | ND | ND | W | India | NGRP | II |
| 116 | PI614440 | *agrestis* | ND | ND | W | India | NGRP | II |
| 117 | PI614441 | *agrestis* | ND | ND | W | India | NGRP | II |
| 118 | PI614442 | *agrestis* | ND | ND | W | India | NGRP | II |
| 119 | PI614479 | *agrestis* | ND | ND | W | India | NGRP | II |
| 120 | PI614481 | *agrestis* | ND | ND | W | India | NGRP | III |
| 121 | PI614486 | *agrestis* | ND | ND | W | India | NGRP | II |
| 122 | PI614493 | *agrestis* | ND | ND | W | India | NGRP | II |
| 123 | PI614494 | *agrestis* | ND | ND | W | India | NGRP | II |
| 124 | PI614514 | *agrestis* | ND | ND | W | India | NGRP | II |
| 125 | PI614519 | *agrestis* | ND | ND | W | India | NGRP | II |
| 126 | PI614525 | *agrestis* | ND | ND | W | India | NGRP | II |
| 127 | PI614526 | *agrestis* | ND | ND | W | India | NGRP | II |
| 128 | PI614558 | *agrestis* | ND | ND | W | India | NGRP | II |
| 129 | PI614565 | *agrestis* | ND | ND | W | India | NGRP | II |
| 130 | PI614573 | *agrestis* | ND | ND | W | India | NGRP | II |
| 131 | PI614574 | *agrestis* | ND | ND | W | India | NGRP | II |
| 132 | PI614575 | *agrestis* | ND | ND | W | India | NGRP | II |
| 133 | PI140471 | *agrestis* | ND | ND | W | The US | NGRP | IV |
| 134 | Mapao-1 | *agrestis* | ND | ND | W | Henan, China | RGWM | IV |
| 135 | Mapao-2 | *agrestis* | ND | ND | W | Henan, China | RGWM | IV |
| 136 | Baipikekouqi | *melo* | *inodorus* | TC | L | Sinkiang, China | NMGWM | III |
| 137 | Furong | *melo* | *cantalupensi* | TC | L | Sinkiang, China | NMGWM | III |
| 138 | Jinbangzi | *melo* | *cantalupensis* | TC | L | Sinkiang, China | NMGWM | III |
| 139 | Malasiguadan | *melo* | *chandalak* | TC | L | Sinkiang, China | NMGWM | III |
| 140 | Baibadeng | *melo* | *inodorus* | TC | L | Sinkiang, China | NMGWM | III |
| 141 | 72-2-3 | *melo* | *inodorus* | TC | L | Sinkiang, China | NMGWM | III |
| 142 | Green Skin Crisp | *melo* | *inodorus* | TC | L | Sinkiang, China | NMGWM | III |
| 143 | Large Planter | *melo* | *cantalupensis* | TC | L | Sinkiang, China | NMGWM | III |
| 144 | Horneseoneen | *melo* | *reticulatus* | TC | L | Henan, China | NMGWM | III |
| 145 | PI164610 | *melo* | *chandalak* | TC | L | India | NGRP | IV |
| 146 | PI163206 | *melo* | *chandalak* | TC | L | India | NGRP | IV |
| 147 | PI164996 | *melo* | *reticulatus* | TC | L | Tunisia | NGRP | III |
| 148 | PI177351 | *melo* | *reticulatus* | TC | L | Tunisia | NGRP | III |
| 149 | PI171599 | *melo* | *reticulatus* | TC | L | Tunisia | NGRP | III |
| 150 | PI381765 | *melo* | *cantalupensis* | TC | L | India | NGRP | III |
| 151 | Aizi | *melo* | *cantalupensis* | TC | L | Sinkiang, China | NMGWM | III |
| 152 | Bayixiangli | *melo* | *inodorus* | TC | L | Sinkiang, China | NMGWM | III |
| 153 | Qiaerkehong | *melo* | *reticulatus* | TC | L | Sinkiang, China | NMGWM | III |
| 154 | Kangbaier | *melo* | *ameri* | TC | L | Sinkiang, China | NMGWM | III |
| 155 | Wangwenmelon | *melo* | *reticulatus* | TC | L | Sinkiang, China | NMGWM | III |
| 156 | Hetaodonggua | *melo* | *chandalak* | TC | L | Inner Mongolia, China | NMGWM | III |
| 157 | PI164409 | *melo* | *reticulatus* | TC | L | India | NGRP | III |
| 158 | PI169320 | *melo* | *adana* | TC | L | Tunisia | NGRP | III |
| 159 | Xinyintian13 | *melo* | *cantalupensis* | TC | L | Japan | NMGWM | III |
| 160 | PI165516 | *melo* | *reticulatus* | TC | L | India | NGRP | III |
| 161 | PI145594 | *melo* | *reticulatus* | TC | L | India | NGRP | III |
| 162 | PI169329 | *melo* | *chandalak* | TC | L | Tunisia | NGRP | III |
| 163 | Jingfeng2610 | *melo* | *cassaba* | TC | L | Henan, China | NMGWM | III |
| 164 | PI163208 | *melo* | *chandalak* | TC | L | India | NGRP | III |
| 165 | Jinmali | *melo* | *chandalak* | TC | L | Gansu, China | NMGWM | III |
| 166 | PI166190 | *melo* | *chandalak* | TC | L | India | NGRP | III |
| 167 | Klakesai | *melo* | *inodorus* | TC | L | Sinkiang, China | NMGWM | III |
| 168 | Houroudonggua | *melo* | *cantalupensis* | TC | L | Sinkiang, China | NMGWM | III |
| 169 | Xinyintian17 | *melo* | *reticulatus* | TC | L | Japan | NMGWM | IV |
| 170 | 20KH.111 | *melo* | *flexuosus* | TC | L | Afghanistan | NMGWM | III |
| 171 | PI137852 | *melo* | *cantalupensis* | TC | L | Iran | NGRP | III |
| 172 | PI140637-1 | *melo* | *cantalupensis* | TC | L | Iran | NGRP | III |
| 173 | PI140815 | *melo* | *chandalak* | TC | L | Iran | NGRP | III |
| 174 | PI163219 | *agrestis* | *ND* | ND | W | India | NGRP | II |
| 175 | PI164852 | *melo* | *chandalak* | TC | L | India | NGRP | III |
| 176 | Meiganma241 | *melo* | *cantalupensis* | TC | L | Gansu, China | NMGWM | III |
| 177 | 92004 | *melo* | *cantalupensis* | TC | L | Iran | NGRP | III |
| 178 | PI140678-S1 | *melo* | *cantalupensis* | TC | L | Iran | NGRP | III |
| 179 | PI165508 | *melo* | *cantalupensis* | TC | L | India | NGRP | III |
| 180 | PI164320-S1 | *melo* | *inodorus* | TC | L | India | NGRP | III |
| 181 | PI164569 | *melo* | *reticulatus* | TC | L | India | NGRP | IV |
| 182 | PI169358 | *melo* | *cantalupensis* | TC | L | Tunisia | NGRP | III |
| 183 | PI143215(1) | *melo* | *ameri* | TC | L | Iran | NGRP | III |
| 184 | PI136223 | *melo* | *chandalak* | TC | L | Iran | NGRP | III |
| 185 | Oujin | *melo* | *chandalak* | TC | L | Beijing, China | NMGWM | III |
| 186 | 87xuanan1 | *melo* | *chandalak* | TC | L | Henan, China | NMGWM | III |
| 187 | Yinxiang | *melo* | *chandalak* | TC | L | Henan, China | NMGWM | III |
| 188 | Fengxuan3 | *melo* | *cantalupensis* | TC | L | Henan, China | NMGWM | IV |
| 189 | Xiantiangua | *melo* | *reticulatus* | TC | L | Henan, China | NMGWM | III |
| 190 | Fenghuang | *melo* | *chandalak* | TC | L | Taiwan, China | NMGWM | III |
| 191 | Lutian2 | *melo* | *chandalak* | TC | L | Shandong, China | NMGWM | III |

TC: Thick-skinned type; TN: Thin-skinned type; ND: not determined.

C: commercial cultivar; L: landrace; W: wild type.

NGRP: USDA-ARS National Genetic Resources Program; NMGWM: National Mid-term Genebank for Watermelon and Melon (Zhengzhou, China); RGWM: Research Group of Watermelon and Melon at Henan Agricultural University.

I, II, III, and IV mean the cluster assignment of the accessions as showed in FIGURE 1.

**Table S2** The information of the 37 SSR markers used in the present study.

| Locus | Chr. | Position (Mb) | Forward primer (5'-3') | Reverse primer (5'-3') | Source |
| --- | --- | --- | --- | --- | --- |
| CM07 | 1 | 0.86 | TTTCCCGCATTGATTTTCTC | GAGAAACGCTTCCCACAAAC | DL |
| CMCT505 | 1 | 16.53 | GACAGTAATCACCTCATCAAC | GGGAATGTAAATTGGATATG | DL |
| SSR011330 | 1 | 30.63 | ACCCATATCCAACTCTCCCC | TGAAGAAATGGGTTTGGAGG | ZH |
| SSR012562 | 2 | 4.14 | TGTTTTCATAGGGGACCTGG | AACATGGCTAAGAGCAGCGT | ZH |
| gSSR4959 | 2 | 6.43 | AACCCAAACTCAAACTCAAAC | GGGAGGAAGAAGAGGAAATA | RG |
| SSR013487 | 2 | 16.44 | CTGCCCCTTTTTCCTTCTCT | ATTGGCCTTTGTTTTCATGG | ZH |
| SSR014660 | 3 | 1.31 | CTTCTCACATGCTTCAACGC | CCCCATTCCCATTTCTTTTT | ZH |
| SSR015784 | 3 | 16.68 | TTAATCTTGCGGTGGAAAGG | TTAGGGAAGGCAATCAATCG | ZH |
| SSR016829 | 3 | 23.89 | CACCCTTCCTTACCAGGACA | GATGCTGTCTTTTTCGTCCC | ZH |
| HNM33 | 4 | 1.05 | TTGCTTCTGCTTCGGACCTT | GGGAGATTTGAATGGTGGTG | RG |
| HNM12 | 4 | 13.37 | ATCTTCTGTCTTGGCCTCCG | GAAAGGGGATGAGTAAAAGTTGAG | RG |
| SSR020162 | 4 | 26.32 | GGGATCATGCATGGGTAAAC | AGCCCTCCAAATATGGCTAA | ZH |
| SSR020947 | 5 | 0.10 | TTTACGCAAGGATGAAAGGG | AATGGGCGAATTAAGAAGCA | ZH |
| DE1557 | 5 | 10.83 | CAAAGACATAAGCCCGATG | AAAAGAAAGATACAAGTTAGGGC | DL |
| SSR023138 | 5 | 24.98 | GGAGGACGAAAGACCAATGA | CGACCGCCATTAATCAAAAC | ZH |
| HNM41 | 6 | 4.57 | TCTTTCCCATAATGGCCTCAG | AACTTGGCTTGGACAGGGAT | RG |
| DE1103 | 6 | 12.97 | CACATGACTTTTCACAAACG | GAATTCTATCTCTGTCTATCAAAG | DL |
| CMAGN52 | 6 | 31.71 | CCACCAACATAACACACAAC | CTCTCACACTGTTGGGAAGA | DL |
| CMAGN75 | 7 | 2.402 | TGGGTTTTCTTCTACTACTG | TGCTTTTACTCTCATTCAAC | DL |
| gSSR22419 | 7 | 12.32 | TTTAGGGTTTAGGGGCTAATA | TTCATGAATTTAGGACACAT | RG |
| SSR029474 | 7 | 24.01 | AACCGCAAATACGAGACCTG | TCTCCTGCATAAACCCCAAG | ZH |
| SSR029716 | 8 | 0.44 | GCGAAATGATTCCATGTTTG | TCCACTTGCTATCTTCTCTCCA | ZH |
| HNM31 | 8 | 11.15 | GCCGTTCTGTGCTTCTTCATT | TTCCGACTCCGTATGTTCAATC | RG |
| HNM40 | 8 | 20.88 | TGCAATTAAAACTTCCACCAAA | TACAACCGTCGCCGTCCT | RG |
| CMTC47 | 9 | 1.70 | GCATAAAAGAATTTGCAGAC | AGAATTGAGAAGAGATAGAG | DL |
| SSR033639 | 9 | 11.46 | AGCAGTGGTAGCAGCAGTAGC | AAGTTTTTCCTTCCCCAGGA | ZH |
| CMATN22 | 9 | 23.38 | CGGCAATCATCTTATCTTTC | AAGATTGAAGTGGGAAAATG | DL |
| CM38 | 10 | 3.95 | TAGCATCTGATCGGAAAACC | CAACTTCATCCGCCAAGAAT | DL |
| CMTCN8 | 10 | 9.99 | CCTCCGCCACATATTACAAT | TTCATCTTGACACGTAAGAG | DL |
| HSSR010 | 10 | 17.65 | GAGGTGGAGGATAAAACAAATG | GATGCTGATAGGAATACCGAGA | RG |
| DM0673 | 11 | 0.56 | TCTACGGACCATGTGAATC | TCAAACAAAGTTGAAATTAGG | DL |
| SSR038372 | 11 | 17.08 | GAAAAGAGTTAGGCTTCCCAA | TCCATTCTCTCTTCCCTGGAT | ZH |
| CMGA104 | 11 | 28.77 | TTACTGGGTTTTGCCGATTT | AATTCCGTATTCAACTCTCC | DL |
| SSR040314 | 12 | 4.42 | TTTGCCACATCATCATGAAA | CCCATGTTTAAGTGATTCCCA | ZH |
| SSR041311 | 12 | 17.75 | CTTCCAACATTCCATTGGCT | TGAAAAGAGATGGGAAACTTTAGTG | ZH |
| CMGAN80 | 12 | 25.80 | ATATTGATTGCTGGGAAAGG | CTTTTTTGGCTTTATTGGGTC | DL |

DL and ZH mean the markers collected from the linkage map of Diaz *et a*l. (2011) and the report of Zhu *et al*. (2016), respectively. RG means the markers developed by our research group.
